# Supplementary material for: Expanding continual few-shot learning benchmarks to include recognition of specific instances
Source: PLoS One. 2024 Jul 5;19(7):e0305856. doi: 10.1371/journal.pone.0305856 (PMC11226023; doi:10.1371/journal.pone.0305856)
Supplement: S1 File — (PDF) [file pone.0305856.s001.pdf]

S1 Methods

1 CFSL framework baseline bugfixes

The two fixes related to weight updates and data processing are described below. The code changes are contained in pull requests by user 'abdel' at https://github.com/AntreasAntoniou/FewShotContinualLearning/pulls?q=is%3Apr+is%3Aclosed.

Weight update The operation which makes a copy of the latest classifier weights is inside the support set loop, rather than the task loop. As a result, affected models will be overridden in every support set, and the target set will be evaluated only by weight updates of the last support set.

Data processing There were two issues related to data processing. The random instance sampling was outside the inner CCI loop resulting in the support sets having the same instances when using CCI>1. Also, the code did not account for class change interval when setting the labels in a task and when calculating the number of output units in a model.

2 Pretrain+Tune method

Unlike the standard configurations of VGG, there are a variable number of blocks, determined by hyperparameters. Additionally, the number of filters increases in each subsequent block similar to the VGG architecture. However, they increase linearly instead of exponentially, i.e., (32-64-96) rather than (32-64-128). Hyperparameters control the number of blocks and filters, as well as the initial learning rate.
