# Supplementary material for: Expanding continual few-shot learning benchmarks to include recognition of specific instances
Source: PLoS One. 2024 Jul 5;19(7):e0305856. doi: 10.1371/journal.pone.0305856 (PMC11226023; doi:10.1371/journal.pone.0305856)
Supplement: S2 File — (PDF) [file pone.0305856.s002.pdf]

S2 Results

1 Scaling test

The scaling test results that are shown with plots in the main body of the manuscript, are shown here in tabular format, Table 1 and Table 2.

Table 1: **Scaling test accuracy – Omniglot.** Results for the best configurations found through hyperparameter search. Accuracy is shown in %, as mean ± standard deviation across 5 random seeds. NC=number of classes.

**Method name**	**Baseline 1** NSS=4, CCI=2, n-way=5, NC=10	**Baseline 2** NSS=8, CCI=2, n-way=5, NC=20	**Wide 1** NSS=4, CCI=2, n-way=10, NC=20	**Wide 2** NSS=4, CCI=2, n-way=100, NC=200	**Deep 1** NSS=20, CCI=2, n-way=2, NC=20	**Deep 2** NSS=80, CCI=2, n-way=5, NC=200
Pretrain+Tune	64.95 ± 1.00	33.71 ± 3.54	54.25 ± 0.59	4.22 ± 0.50	33.44 ± 1.18	6.64 ± 0.68
Pretrain+Tune+Replay	81.15 ± 0.81	73.5 ± 0.43	80.74 ± 0.70	31.78 ± 0.65	60.94 ± 0.90	18.62 ± 0.71
ProtoNets	86.67 ± 1.35	88.04 ± 1.12	86.92 ± 0.42	65.61 ± 9.31	88.56 ± 0.61	80.30 ± 1.15

Table 2: **Scaling test accuracy – SlimageNet64.** Results for the best configurations found through hyperparameter search. Accuracy is shown in %, as mean ± standard deviation across 5 random seeds. NC=number of classes.

**Method name**	**Baseline 1** NSS=4, CCI=2, n-way=5, NC=10	**Baseline 2** NSS=8, CCI=2, n-way=5, NC=20	**Wide 1** NSS=4, CCI=2, n-way=10, NC=20	**Wide 2** NSS=4, CCI=2, n-way=100, NC=200	**Deep 1** NSS=20, CCI=2, n-way=2, NC=20	**Deep 2** NSS=80, CCI=2, n-way=5, NC=200
Pretrain+Tune	15.85 ± 0.20	7.80 ± 0.10	9.40 ± 0.19	4.83 ± 0.05	5.89 ± 0.01	2.66 ± 0.02
Pretrain+Tune+Replay	13.59 ± 0.14	9.79 ± 0.16	10.45 ± 0.18	4.76 ± 0.04	9.35 ± 0.08	4.28 ± 0.06
ProtoNets	25.72 ± 0.17	18.24 ± 0.15	18.04 ± 0.10	11.62 ± 0.04	19.29 ± 0.16	12.21 ± 0.10

2 Optimised hyperparameters

To facilitate fairer comparison of architectures, hyperparameter optimisation was used to find the best configuration under each experimental condition. The resulting hyperparameter values used in experiments are shown in Table 3 and Table 4.

Table 3: **Scaling test, best hyperparameters – Omniglot.** Results for the best architectures found for each experiment, selected through hyperparameter search. A block consists of a 2d convolutional layer, a batch norm layer and a max pooling layer. The number of filters is for the first block, and it increases linearly for each subsequent block. The learning rate is also shown, denoted with lr. Unless specified, lr=0.01. In the case of the replay buffer, b denotes the size of the buffer in support sets, and p denotes the number of samples taken from the buffer for each fine-tuning support set.

**Method name**	**Baseline 1** NSS=4, CCI=2, n-way=5, NC=10	**Baseline 2** NSS=8, CCI=2, n-way=5, NC=20	**Wide 1** NSS=4, CCI=2, n-way=10, NC=20	**Wide 2** NSS=4, CCI=2, n-way=100, NC=200	**Deep 1** NSS=20, CCI=2, n-way=2, NC=20	**Deep 2** NSS=80, CCI=2, n-way=5, NC=200
Pretrain+Tune	128 filters, 3 blocks	512 filters, 3 blocks	256 filters, 3 blocks	128 filters, 2 blocks	256 filters, 3 blocks	256 filters, 3 blocks
Pretrain+Tune+Replay	512 filters, 3 blocks, b=2, p=10	128 filters, 3 blocks, b=4, p=10	256 filters, 3 blocks, b=2, p=20	128 filters, 2 blocks, b=2, p=50	256 filters, 3 blocks, b=5, p=10	256 filters, 3 blocks, b=5, p=10
ProtoNets	128 filters, 4 blocks	128 filters, 4 blocks, lr=0.001	128 filters, 4 blocks, lr=0.001	128 filters, 4 blocks	128 filters, 4 blocks	256 filters, 4 blocks, lr=0.001

Table 4: **Scaling test, best hyperparameters – SlimageNet64.** Results for the best architectures found for each experiment, selected through hyperparameter search. A block consists of a 2d convolutional layer, a batch norm layer and a max pooling layer. The number of filters is for the first block, and it increases linearly for each subsequent block. In all cases, the learning rate, lr=0.01. In the case of the replay buffer, b denotes the size of the buffer in support sets, and p denotes the number of samples taken from the buffer for each fine-tuning support set.

**Method name**	**Baseline 1** NSS=4, CCI=2, n-way=5, NC=10	**Baseline 2** NSS=8, CCI=2, n-way=5, NC=20	**Wide 1** NSS=4, CCI=2, n-way=10, NC=20	**Wide 2** NSS=4, CCI=2, n-way=100, NC=200	**Deep 1** NSS=20, CCI=2, n-way=2, NC=20	**Deep 2** NSS=80, CCI=2, n-way=5, NC=200
Pretrain+Tune	64 filters, 4 blocks	64 filters, 4 blocks	64 filters, 4 blocks	128 filters, 4 blocks	64 filters, 4 blocks	64 filters, 4 blocks
Pretrain+Tune+Replay	256 filters, 4 blocks, b=2, p=20	64 filters, 4 blocks, b=2, p=20	64 filters, 4 blocks, b=8, p=10	128 filters, 4 blocks, b=8, p=12	64 filters, 4 blocks, b=2, p=5	64 filters, 4 blocks, b=4, p=10
ProtoNets	64 filters, 4 blocks	64 filters, 4 blocks	64 filters, 4 blocks	64 filters, 4 blocks	128 filters, 4 blocks	64 filters, 4 blocks

3 Fine-tuning steps

Adding a replay buffer increased the memory requirements. For some experiments, we reduced the number of fine-tuning training steps to make it possible to run within our hardware constraints. The number of steps are documented in Tables 5 and 6.

Table 5: **Fine-tuning for scaling test.** The number of fine-tuning training steps for the Pretrain+Tune+Replay scaling test.

**Experiment**	**Fine-tuning training steps**
Baseline 1	120
Baseline 2	60
Wide 1	30
Wide 2	30
Deep 1	5
Deep 2	5

Table 6: **Fine-tuning for the instance test.** The number of fine-tuning training steps for the Pretrain+Tune+Replay instance test.

**Experiment**	**Fine-tuning training steps**
Exp. 1	120
Exp. 2	120
Exp. 3	60
Exp. 4	30
