# Supplementary material for: Expanding continual few-shot learning benchmarks to include recognition of specific instances
Source: PLoS One. 2024 Jul 5;19(7):e0305856. doi: 10.1371/journal.pone.0305856 (PMC11226023; doi:10.1371/journal.pone.0305856)
Supplement: Attachment — Submitted filename: Response to Reviewers.pdf [file pone.0305856.s004.pdf]

Thank you to the reviewers for taking the time to read the paper and provide valuable feedback. In response, we made many changes which have improved the paper. Please see reviewer comments in blue and our responses below in black, with excerpts from the paper indented and changes in purple. Please also refer to the marked up copy of the manuscript that highlights changes, named 'Revised Manuscript with Track Changes.pdf'.

1. Can you elaborate on how the parameters (NSS, CCI, n-way, k-shot) are chosen for each experiment?

Thanks for the suggestion. It was not explained enough. We updated the existing introduction of these parameters by labelling them as 'experiment hyperparameters' and then referred to them in subsequent subsections.

> The experiment is parameterised by a small set of 'experiment hyperparameters' described in Table I. By varying these parameters, the experimenter can flexibly control the few-shot continual learning tasks, the total number of classes (NC), samples per class, and the manner in which they are presented to the learner.

For CFSL at scale, see below:

> **B. CFSL at scale**
>
> We chose to base our experiments on the *experiment hyperparameters* of Task D (see Figure 1) as described in the original CFSL benchmark (Antoniou 2020) as it resembles the most common and applicable real-world scenario -- it introduces both new classes and additional instances of each class.

And then in the relevant method subsections, we added descriptions:

> **B 1) Framework baseline -- replicating the original experiments**
>
> Given the significance of some of these issues, we opted to replicate a selected number of the original experiments *(by setting the same experiment hyperparameters i.e. NSS, CCI, k-shot and n-way)* to properly contextualise our new experiments and results.
>
> **B 2) Scaling**
>
> Two of the replication configurations were used as baselines, with 10 and 20 classes. Then, we created Wide and Deep configurations, with 20 total classes like the 2nd baseline, but we modified the way that the classes were presented. Finally, the number of classes was increased ten-fold to 200, presented in both Wide and Deep configurations. In all of the experiments, k-shot is set to 1, so for any support set, there is only one exemplar per class. *The other experiment hyperparameters (NSS, CCI and n-way) were chosen to achieve the desired total number of classes (NC) and the Wide and Deep configurations described above (see Figure 2 for an illustration of how they can be used to manipulate support set size and number of tasks).*

And for the Instance Test, we draw your attention to the following paragraph, which explains the choice of experiment hyperparameters.

> Due to the flexibility of the CFSL framework, the instance test can be implemented as a special case of the existing parameters. n-way is set to 1, so that there is only one class in each support set. CCI is equal to NSS so that there is no class change between support sets. Then the k-shot or samples per class, determines how many instances are shown for a given class, which we refer to as Number of Instances, NI. We used a constant total number of instances for all experiments, 20, but experimented with presenting the samples differently, in terms of number and size of support sets. We reused empirically optimal hyperparameters from the Scaling Experiments.

2. Could you provide more details on the issues identified in the original CFSL codebase and how they were addressed?

It's a good idea to include a detailed explanation. We therefore added the following Appendix section.

> **CFSL framework baseline bugfixes**
>
> The two fixes related to weight updates and data processing.
> *a) Weight update:* The operation which makes a copy of the latest classifier weights is inside the support set loop, rather than the task loop. As a result, affected models will be overridden in every support set, and the target set will be evaluated only by weight updates of the last support set.
>
> *b) Data processing:* There were two issues related to data processing. The random instance sampling was outside the inner CCI loop resulting in the support sets having the same instances when using CCI$>$1. Also, the code did not account for class change interval when setting the labels in a task and when calculating the number of output units in a model.
>
> The code changes are contained in pull requests by user `abdel' at https://github.com/AntreasAntoniou/FewShotContinualLearning/pulls?q=is%3Apr+is%3Aclosed.

3. Can you elaborate on how the instance test was designed to reflect real-world challenges, such as noise and occlusion?

Thank you for the question, the text was missing important motivation. We expanded and modified the section on the Instance Test. See first 2 paragraphs below.

> As discussed above, we seek to increase the range of natural conditions and challenges that are captured by the CFSL benchmark, to include recognition of a specific single object -- one instance of a class. We call this the 'instance test', based on the instance test in AHA (Kowadlo 2020). The learner must learn to recognize specific exemplars amongst sets where

> all the exemplars are drawn from the same class. *An important feature of the instance test is the addition of increasing levels of corruption, intended to resemble real-world conditions and imperfect sensor readings. White noise models scenarios such as where the object is dirty, colour changed or there are varying light conditions. Occlusion models incomplete sensing, most commonly due to partial obstruction by another object.*
>
> *The instance test is not simply trying to find duplicates, since the varying levels of random corruption (noise and occlusion) are applied.* It is a setting that has been largely ignored in ML, and yet it represents many important real world situations. For example, a person can easily identify their own specific cup of coffee, or their own locker, and not see them simply as a generic cup or locker class*, despite those objects appearing differently due to occlusion and lighting changes*. For a tangible robotics example, take a pick-and-place robot that should place items in a specific bin (rather than any generic bin).

4. Are there any insights into why ProtoNets were less disrupted by noise and occlusion in the SlimageNet64 dataset compared to the Omniglot dataset?

We agree this was an interesting phenomena. We discussed it in the Discussion, subsection 'C. Specific instances (no replay)'. Please see it reproduced below. We moved it into its own paragraph, to make it more prominent. See below.

> ProtoNets were less disrupted by noise and occlusion in the SlimageNet64 dataset, probably because these images have background content which provides stable features that allow recognition. The desirability of using background features for recognition depends on the intent of the task. In some cases, recognising the scene where an object was placed might be the only way to distinguish it.

We felt it was best placed in this section because it is focussed on the instance test, but could move it to the first subsection dedicated to comparing Pre-train+Tune and ProtoNet methods.

5. It is essential to ensure that the literature review encompasses the most recent advancements in the field.

Thankyou for pointing this out. We improved the literature review with recent advancements covering many additional papers. The new section is large, so please refer to the new uploaded paper, Section II.C.
