# Supplementary material for: Expanding continual few-shot learning benchmarks to include recognition of specific instances
Source: PLoS One. 2024 Jul 5;19(7):e0305856. doi: 10.1371/journal.pone.0305856 (PMC11226023; doi:10.1371/journal.pone.0305856)
Supplement: Attachment — Submitted filename: review comment 23.3.24.docx [file pone.0305856.s003.docx]

1. Can you elaborate on how the parameters (NSS, CCI, n-way, k-shot) are chosen for each experiment?
2. Could you provide more details on the issues identified in the original CFSL codebase and how they were addressed?
3. Can you elaborate on how the instance test was designed to reflect real-world challenges, such as noise and occlusion?
4. Are there any insights into why ProtoNets were less disrupted by noise and occlusion in the SlimageNet64 dataset compared to the Omniglot dataset?
5. It is essential to ensure that the literature review encompasses the most recent advancements in the field.
